# Lessons learned from conducting six multi-country mixed-methods effectiveness research studies on water, sanitation, and hygiene (WASH) interventions in humanitarian response

**DOI:** 10.1186/s12889-021-10597-z

**Published:** 2021-03-22

**Authors:** Daniele Lantagne, Lilian Lehmann, Travis Yates, Karin Gallandat, Mustafa Sikder, Marta Domini, Gabrielle String

**Affiliations:** 1 Tufts University School of Engineering, Medford, MA USA; 2 Innovations for Poverty Action, New Haven, CT USA; 3 IDinsight, Manila, Philippines; 4 London School of Hygiene and Tropical Medicine, London, UK; 5 Johns Hopkins University, Center for Humanitarian Health, Baltimore, MD USA; 6 Brescia University, Brescia, Italy

**Keywords:** Effectiveness research, Ethics, Humanitarian response, Operational research, Water, sanitation and hygiene

## Abstract

**Background:**

Provision of safe water, sanitation, and hygiene (WASH) to affected populations in humanitarian emergencies is necessary for dignity and communicable disease control. Additional evidence on WASH interventions is needed in humanitarian settings. Between 2008 and 2019, we completed six multi-country, mixed-methods effectiveness studies in humanitarian response on six different WASH interventions. In each evaluation, we conducted: key informant interviews; water point observations and water quality testing; household surveys with recipients, including survey and water quality testing; focus group discussions; and/or, secondary data analysis. The research questions were: “What is the effectiveness of *[intervention]* in reducing the risk of diarrhea/cholera transmission; and, what programmatic factors lead to higher effectiveness?”

**Discussion:**

In all six multi-country, mixed-methods evaluations, policy-relevant outcomes were obtained. We found, in our individual research results, that: interventions could reduce the risk of disease in humanitarian contexts; this reduction of risk did not always occur, as there were large ranges in effectiveness; and, implementation factors were crucial to intervention effectiveness. When collaboratively reviewing our research process across evaluations, we found strategies for successfully conducting this research included: 1) working with *partners* to identify and evaluate programs; 2) rapidly obtaining approvals to *deploy*; and, 3) conducting research methodologies *consistently*. Personal connections, in-person communication, trust, and experience working together were key factors for success in identifying partners for evaluation. Successes in evaluation deployment occurred with flexibility, patience, commitment of adequate time, and understanding of processes; although we note access and security concerns in insecure contexts precluded deployment. Consistent and robust protocols, flexibility, and a consistent researcher on the ground in each context allowed for methodological consistency and high-quality results.

**Conclusions:**

In conclusion, we have found multi-country, mixed-methods results to be one crucial piece of the WASH evidence base in humanitarian contexts. This is particularly because evaluations of reductions in risk from real-world programming are policy-relevant, and are directly used to improve programming. In future, we need to flexibly work with donors, agencies, institutions, responders, local governments, local responders, and beneficiaries to design safe and ethical research protocols to answer questions related to WASH interventions effectiveness in humanitarian response, and, improve WASH programming.

## Background

Humanitarian emergencies are occurring at increasing rates and affecting greater numbers of people [1–3]. Provision of safe water, sanitation, and hygiene (WASH) to affected populations is necessary for human dignity and communicable disease control [4–6]. WASH interventions that have been evaluated consistently reduce the risk of disease transmission, and risk of disease, in humanitarian emergencies [7, 8]. Program design, community engagement, and beneficiary preferences are important factors to ensure effectiveness.

Overall, there is need to use existing evidence to strengthen policy and practice [7, 8], while simultaneously strengthening the evidence base [9]. Methodologies recommended for strengthening the evidence base are: 1) testing interventions in the laboratory for *efficacy* in ideal circumstances across defined test conditions; 2) field testing using multi-country, mixed-methods evaluations to determine if (and under what conditions) interventions can be *effective;* and, 3) for efficacious and effective interventions, conducting *health impact* and *cost-effectiveness* evaluations [10].

The goal of multi-country, mixed-methods research is to determine effectiveness by evaluating the same intervention, using the same methodology, across different contexts. The research questions asked in our studies were: “What is the effectiveness of *[intervention]* in reducing the risk of diarrhea/cholera transmission; and, what programmatic factors lead to higher effectiveness?”

To date, we have completed six multi-country, mixed-methods effectiveness studies in humanitarian response (Table 1), on: 1) distributions of household water treatment (HWT) [11]; 2) installation of source-based chlorination water treatment (Dispensers) [12]; 3) development of Water Safety Plans (WSPs) [13]; 4) spraying household surfaces with chlorine to prevent ongoing transmission of cholera (household spraying) [14]; 5) trucking water to an affected population (water trucking) [15]; and, 6) stationing a worker dispensing chlorine into water collection containers at water sources (bucket chlorination) (*manuscript in preparation*).

**Table 1:** Information on six multi-country, mixed-methods evaluations

	HWT	Dispensers	Water Safety Plans	Household Spraying	Water Trucking	Bucket Chlorination
Years Data Collected	2008–2010	2011–2013	2015–2017	2017–2019	2017–2019	2017–2019
Donor	UNICEF Oxfam	BMGF	UNICEF	R2HC	OFDA	OFDA/R2HC
Countries evaluated	Haiti Kenya Indonesia Nepal	DRC Haiti Sierra Leone Senegal	DRC Fiji India Vanuatu	DRC Haiti Mozambique	CXB DRC	CXB DRC Haiti Mozambique Nigeria
Emergency type	Acute: earthquakes, cholera, flooding	Acute: cholera, food insecurity	^a^	Endemic and epidemic cholera settings	Long-term, recurring, refugee	Recurring, long-term contexts and acute outbreak
Criteria for evaluation	HWT in acute	Criteria defined in initial meeting	WSP with UNICEF	Any implemented program		
Partners (planned)	Determined after arrival	Set in grant, provided funding for implementation	UNICEF partners	Pre-selected partners, also open to all	Open, any OFDA country	Pre-selected and open to any OFDA countries
Ethics (Institutional)	Pre-approval at LSHTM	Pre-approval at IPA	SBER pre-review of protocol and tools, modifications submitted to Committee by country, re-reviewed, final approval after local approval via formal SBER review process or review or (if not available/appropriate) by qualified personnel who provided letter obtained			
Ethics (local)	Not required during this time	Not required during this time				
Research budget (USD)	~ 300,000	~ 800,000	~ 150,000+	~ 90,000	~ 100,000	~ 190,000

^a^ Study was not originally specific to humanitarian contexts, however, DRC was a long-term emergency, Fiji/Vanuatu recovering from acute emergencies, and Fiji/India/Vanuatu contexts planning for future climate emergencies

Effectiveness was defined as measures of *risk reduction* using consistent outcomes (e.g. reductions of *E. coli* in stored household water, reductions of *V. cholerae* on household surfaces, knowledge gained by beneficiaries, and/or meeting international standards for water quantity/quality). Please note these outcomes are not direct health impacts; interventions with successful risk reduction could be further evaluated for health impact.

The mixed-methods tools used in the evaluations were consistent within each evaluation and included: 1) key informant interviews with program managers, implementers, and staff, followed by qualitative analysis; 2) water point observations and water quality testing; 3) initial and/or follow-up household surveys with recipients, including water quality testing for free chlorine residual (FCR) and *E. coli* of stored untreated and treated waters, or surfaces for *V. cholerae*; 4) focus group discussions with beneficiaries; and/or, 4) secondary analysis of program monitoring and/or cost data.

In all six multi-country, mixed-methods evaluations, policy-relevant outcomes were obtained. We found: interventions could reduce the risk of disease in humanitarian contexts; this reduction of risk did not always occur, as there were large ranges of effectiveness; and, implementation factors were crucial to intervention effectiveness [11–15] (*manuscripts in preparation*). Please note that, to generate this manuscript of lessons learned from the process of conducting multi-country, mixed-methods evaluations, the lead investigators and researchers of the six studies met, discussed, and wrote up these lessons learned collaboratively.

## Discussion

### Scientific importance of research

The scientific importance of multi-country, mixed-methods research is in evaluating actual WASH interventions in real time in real-world settings, which are often very different from controlled, laboratory settings. Additionally, real-world settings include evaluations of actual humanitarian response programming, which may be different from development or routine programming. Findings are then disseminated through peer-reviewed manuscripts, reports to donors/partners within 30–60 days, implementation manuals, conference presentations, technical memorandum, fact sheets, and webinars. Our experience is evaluation results are desired by implementers, as they are directly used to improve programming.

For example, HWT intervention efficacy is well established in the laboratory setting [16], and in development-context health impact studies [17]. Our multi-country, mixed-methods research filled an important gap: is HWT effective in humanitarian response? Results were incorporated into the SPHERE Handbook guidelines for the humanitarian sector as guidance, and with a decision tree; it is now commonly accepted that HWT distribution without beneficiary training is ineffective [18]. Effectiveness studies (unlike laboratory, or some health impact studies) must be conducted in actual humanitarian response programs to: account for contextual factors that influence effectiveness; and, convince results users of the applicability to humanitarian response.

#### Strategies & challenges to research

Because multi-country, mixed-methods research is based on evaluating actual programming across contexts, strategies for conducting the process of this research include: 1) working with *partners* to identify and evaluate programs; 2) rapidly obtaining approvals to *deploy* to evaluation contexts; and, 3) implementing research methodologies *consistently*. Each is further described below.

### Partnerships

Across the six evaluations, different modalities were used to determine evaluation partners (Table 1), including: identifying partners after deployment (HWT), working with pre-selected partners who received sub-contracts to implement programs to be evaluated (Dispensers), identifying partners through donors (WSPs), pre-identifying potential partners while also leaving open evaluations to any partner (household spraying and bucket chlorination), or evaluating any program in a donor-approved country (water trucking and bucket chlorination).

In all cases, the main mechanism to connect researchers to programs to be evaluated was personal connections, often mediated through the United Nations WASH Cluster platform, international agencies such as UNICEF, donors, and personal meetings in informal locations such as conferences and hotels. Challenges included difficulties coordinating among international and local partner office objectives, and identifying appropriate programs with pre-selected partners (in the Dispensers evaluation, one pre-selected partner returned funding because implementation was not possible). Key factors for success in identifying evaluation partners were personal connections, in-person communication, trust, and experience working together. For example, in-person meetings at conferences and personal introductions were critical to identifying programs to evaluate.

### Rapid deployment after emergencies

In 2008, for the HWT evaluation, the deployment process was to: submit an ethics board protocol for pre-approval for four to-be determined contexts; communicate with donors to determine when to deploy; and, deploy to responses, identify partners conducting distributions, finalize study design based on programs implemented, translate and finalize study tools, and complete data collection. On the researchers’ part, this required flexibility, an acceptance of risk in deploying to contexts with unknowns, thinking-on-your-feet adaptation, and the ability to rapidly develop on-the-ground partnerships.

Over the last decade, significant protections have been established for conducting research in humanitarian response. These protections are important, and have research consequences. In 2017–2019 the deployment process was: institutional ethics pre-approval of a template protocol; identification of specific programs to evaluate (as described above in partnerships); and, contacting programs to obtain specific information necessary for approvals at institutional, responder, local, and donor levels, including: *institutional* (security clearance, full ethics review of final evaluation tools); *responder* (ethics review, approval layers); *local government* (ethics approvals, visa, importation of sampling equipment in checked baggage); and, *donor* (approval of deployment). Coordinating this approval process chain required ~ 3 months of researcher time, in addition to responder time.

These approval processes impacted research. For the HWT work, and Dispenser and WSP evaluations, evaluated contexts were diverse, and included acute onset emergencies. In the water trucking, household spraying, and bucket chlorination evaluations, we sought to work within the new approval processes by: 1) developing pre-reviewed ‘shell’ protocols that could be rapidly adapted for final ethics approval; 2) working with an extensive list of contacts (as described above) to continuously monitor responses that might meet inclusion criteria; 3) pre-obtaining local ethics approvals in likely countries; and, 4) having staff available to deploy at all times.

Despite these ameliorations, we were unable to deploy to a number of contexts, including a: cholera outbreak in Niger (local responder did not know local ethics process during short program implementation timeframe); cholera outbreak in DRC (denied by institutional security); household spraying and water trucking during a cholera outbreak in Nigeria (local ethics process takes 6–8 months, longer than program implementation timeframe); water trucking programs in Uganda and other countries (not allowable under donor mandate); and, water trucking in Indonesia and evaluations in Cox’s Bazar (visa restrictions for international researchers/responders). Overall, context diversity was reduced in water trucking, bucket chlorination, and household spraying evaluations, where approval processes made it challenging to quickly deploy, particularly to acute emergencies. As such contexts evaluated were more stable, accessible, long-term, protracted contexts where crises could be predicted and approval processes begun in advance. Additionally, in three individual evaluations, the approvals processes delayed deployment, and researchers arrived near program end, or, in one case, after program end. The focus on research in stable contexts is a limitation of humanitarian response research [19], as data from those potentially most impacted is not obtained.

Flexibility allowed us to overcome some of these challenges and complete evaluations. For example, a Bangladeshi Ph.D. student in the group completed all evaluations in Cox’s Bazar; bucket chlorination in the Nigeria cholera outbreak was evaluated 1 year later than initially planned in the next cholera season; and, more experienced personnel traveled to more insecure contexts.

While some components of this process evolution seemed crucially important, particularly incorporation of local ethical approvals, other components were process-oriented. For example, institutional distinction between post-doctoral scholars and Ph.D. students in travel clearances is not appropriate, and the fifth institutional ethics review of the same protocol highlights the need for streamlining. All research described herein is minimal risk, and, interestingly (except for one responding organization) we only received administrative comments from ethics review boards, we never received comments related to protecting human subjects.

There are also gaps and unintended consequences in this set of regulatory processes. For example, there is no specific training for ethical review boards for emergency-affected populations, and review does not always incorporate adequate protections for this population, including: the required standard written consent form can intimidate respondents (leading to the question of whether requesting a waiver of consent or verbal consent is more ethically appropriate); there is insufficient consideration of respondent mental health; and, national ethics review boards may not be appropriate to review protocols for refugee populations hosted in their country. Furthermore, emergency-affected populations should be considered vulnerable in ethical review.

Additionally, there is insufficient training related to mental health needs of research staff who work in humanitarian response settings. The personal and mental health impacts on humanitarian responders are well established [20]. However, the cumulative impact of intense, short-term deployments in humanitarian response contexts, and the physical and mental health needs of local staff (who are often part of the affected population), are often not sufficiently addressed. Moreover, while security protocols are well established for international staff, they are often insufficient for local staff.

Overall, lessons learned in deployment came with lack of processes aligning. Successes in deployment occurred with flexibility, patience, commitment of adequate time, and understanding of processes. Additionally, despite increased regulation over the last decade, there are significant gaps in protecting affected populations, researchers, and local staff.

### Methodological challenges and consistency

Across all evaluations, as described above, the benefit of mixed-methods methodology is the use of consistent and systematic research design, activities, tools, and outcomes across contexts. This allows: a broad range of data to be collected; and, data triangulation to generate both research results and general, policy-relevant recommendations.

Furthermore, given the diversity of data collected, if a particular data type could not be collected in a certain context it was not a critical loss. For example: 1) microbiological sampling could not be conducted in the extremely remote data collection site in Nepal-HWT study, FCR data was used instead; 2) informants in KIIs in Cox’s Bazar did not allow recording of interviews, and notes taken during the conversation by the researcher were analyzed; 3) water truckers did not allow the researcher into their truck, and researchers followed trucking routes in a chase car; and, 4) due to security, it was not possible in some contexts to return to households to conduct follow-up visits.

Across all six multi-country, mixed-methods evaluations, the actual systematic, consistent protocol was viewed as a best-case, ideal, and as much of the protocol was completed as was possible given the local contextual factors. In all six examples of multi-country, mixed-methods results, sufficient data to make policy and programmatic recommendations were obtained. While sometimes results were unexpected, or not anticipated by the program/donor; the consistency and diversity of the methodology ensured that each evaluation produced relevant results.

Methodological challenges were also overcome by having a consistent researcher on the ground in each context. In other studies, providing research protocols to different institutions in different locations without significant training and day-to-day oversight from a consistent person led to small day-to-day decision-making that diverged the studies, and comparability and cross-context outcomes and impacts were lost. Thus, it is necessary for consistent research staff presence in the field to make the day-to-day decisions and ensure the details align across contexts.

### Summary

As recipients of limited research dollars for humanitarian response research, it is important to ask what are the most efficient, cost-effective, and ethical means to complete WASH research in humanitarian contexts? To answer this larger question, some questions to ask ourselves are: Who determines the research questions, to ensure that results maximally inform policy and future implementations? Should this research be in the academic space or at responder/NGO level? Should Ph.D. students complete this research or not? What is acceptable risk for a researcher, how is that risk mitigated, and who makes that decision? Is it necessary to fly one (international) researcher into each context to ensure consistency? Could protocols be sent to local institutions and completed locally? What is the role of local capacity building in evaluations of short-term humanitarian response programs? What are alternatives to multi-country, mixed-methods research? If multi-country, mixed-methods research continues to be conducted, what are the pre-requisites to completing it?

As the WASH humanitarian response sector continues to evolve, these questions are being answered. Almost universally, WASH humanitarian response donors are moving toward requiring partnerships between research and response organizations for funded evaluations. Increasingly, and particularly with European donors, partnerships with Southern institutions are also required, and funding for Ph.D. students is unallowable. In research budgets, including funding for trainings, capacity building, and dissemination activities is encouraged. On the positive side, these donor-led decisions reduce the ‘research savior complex’, and lead to more experienced researchers completing research in conjunction with long-term local researchers. On the negative side, the barrier to entry to humanitarian research is higher, and contexts most in need may be left out. While presenting multi-country, mixed-methods results at a conference, a question we received from a qualified, prestigious researcher was “how did you DO this?” When we answered, they replied, “I would like to do this research, but cannot manage all these logistics”.

A common modality for global health research, and humanitarian research in more stable contexts, is for an international academic institution, an international response organization, a local response organization, and a local research university to work together. However, there are large questions about how to complete research in countries: in conflict (e.g. Syria, Yemen); where the local government is not supportive of the affected population (e.g. Myanmar); where unexpected rapid-onset emergencies occur; where local academic institutions have been destroyed, are not able to work with external partners, or do not have the capacity to conduct research; and, where the local approval processes may not adequately protect a community hosted within their borders.

In addition to completing multi-country, mixed-methods evaluations as described herein, our group at Tufts has had success in completing work in contexts that have the potential to be left out of research including: mixed-methods evaluation protocols on interventions within one country (Haiti, Cox’s Bazar), which simplified IRB and security logistics considerably [21]; working with UNICEF and (for security reasons, unnamed) local partners to analyze effectiveness data collected within Syria with enumerators trained over WhatsApp and data sent across the border via cell phone [22, 23]; analyzing pre-existing data and conducting effectiveness research with IDPs in Myanmar, with the support of the WASH Cluster for visas, travel authorizations, and with the WASH Cluster-established ethics approval board (*manuscript in preparation)*; and, establishing long-term research partnerships with specific response organizations. All of these mechanisms limit the scope of the multi-country, mixed-methods work to a logistically manageable subset of contexts. These factors, including alternative but appropriate ethics review processes that protect the local population, long-term research partnerships, remote data collection (including funding for planning trips in long-term contexts), hosting national Ph.D. students in international institutions, and having arrangements with the WASH Cluster/UN system for visa support, are new methods of WASH humanitarian response research likely to increase in the future.

The evolution in humanitarian response research over the last decade was needed, and this evolution will continue into the future. Multi-country, mixed-methods research provides crucial policy-relevant and valuable results, and will have a role as an evaluation tool, particularly in intervention assessment. In the end, as researchers, we hold ourselves accountable to the following question: How do we ethically and safely conduct research that protects the local population and gathers data that improves WASH interventions in humanitarian response? The myriad current regulation systems do not lend themselves to holistically keeping this core question at heart in humanitarian contexts; it is the researcher’s responsibility to do this, while also keeping in mind the importance of conducting research with those most in need.

## Conclusions

In conclusion, we have found multi-country, mixed-methods results to be one crucial piece of the WASH evidence base in humanitarian contexts. This is particularly because evaluations of reductions in risk from real-world programming are policy-relevant, and directly used to improve programming. The question moving into the future is how to complete these evaluations ethically while protecting the safety of all involved. As researchers, we cannot ignore populations most in need because it is difficult to coordinate research in these contexts. We need to flexibly work with donors, agencies, institutions, responders, local governments, local responders, beneficiaries, and local researchers to design systematic, consistent, and robust research protocols and continue to develop methods to answer important questions related to WASH interventions effectiveness in humanitarian response, and, improve WASH programming into the future.

## Data Availability

Data sharing is not applicable to this article as no datasets were generated or analyzed during the current study.

## Abbreviations

BMGF: Bill & Melinda Gates Foundation
CXB: Cox’s Bazar Refugee Camp
DRC: Democratic Republic of Congo
HWT: Household Water Treatment
IDPs: Internally Displaced Persons
IPA: Innovations for Poverty Action
LSHTM: London School of Hygiene and Tropical Medicine
OFDA: Office of Foreign Disaster Assistance
R2HC: Research for Health in Humanitarian Response
USD: United States Dollars
WASH: Water, Sanitation, and Hygiene
WHO: World Health Organization
WSP: Water Safety Plans
